# Rietveld refinement of Sr_5_(AsO_4_)_3_Cl from high-resolution synchrotron data

**DOI:** 10.1107/S1600536809005054

**Published:** 2009-02-21

**Authors:** Anthony M. T. Bell, C. Michael B. Henderson, Richard F. Wendlandt, Wendy J. Harrison

**Affiliations:** a Synchrotron Radiation Source, STFC Daresbury Laboratory, Daresbury, Warrington, Cheshire WA4 4AD, England; b School of Earth, Atmospheric and Environmental Sciences, University of Manchester, Manchester, M13 9PL, England; c Department of Geology and Geological Engineering, Colorado School of Mines, Golden, CO 80401, USA

## Abstract

The apatite-type compound, penta­strontium tris­[arsenate(V)] chloride, Sr_5_(AsO_4_)_3_Cl, has been synthesized by ion exchange at high temperature from a synthetic sample of mimetite [Pb_5_(AsO_4_)_3_Cl] with SrCO_3_ as a by-product. The results of the Rietveld refinement, based on high resolution synchrotron X-ray powder diffraction data, show that the title compound crystallizes in the same structure as other halogenoapatites with general formula *A*_5_(*Y*O_4_)_3_ *X* (*A* = divalent cation, *Y* = penta­valent cation, and *X* = F, Cl or Br) in the space group *P*6_3_/*m*. The structure consists of isolated tetra­hedral AsO_4_^3−^ anions (the As atom and two O atoms have *m* symmetry), separated by two crystallographically independent Sr^2+^ cations that are located on mirror planes and threefold rotation axes, respectively. One Sr atom is coordinated by nine O atoms and the other by six. The chloride anions (site symmetry 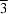) are at the 2*a* sites and are located in the channels of the structure.

## Related literature

For crystal chemistry of apatites, see: Mercier *et al.* (2005 ▶); White & ZhiLi (2003 ▶); Wu *et al.* (2003 ▶). For powder diffraction data on Sr As-apatite, see: Kreidler & Hummel (1970 ▶). Atomic coordinates as starting parameters for the Rietveld (Rietveld, 1969 ▶) refinement of the present phases were taken from Bell *et al.* (2008 ▶); Dai *et al.* (1991 ▶); de Villiers *et al.* (1971 ▶). For related Sr—Cl-apatites, see: Đordević *et al.* (2008 ▶); Sudarsanan & Young, (1974 ▶, 1980 ▶); Beck *et al.* (2006 ▶); Noetzold *et al.* (1995 ▶); Noetzold & Wulff (1996 ▶, 1997 ▶, 1998 ▶); Swafford & Holt (2002 ▶); Wardojo & Hwu (1996 ▶). For synthetic work, see: Baker (1966 ▶); Essington (1988 ▶); Harrison *et al.* (2002 ▶).

## Experimental

### 

#### Crystal data

Sr_5_(AsO_4_)_3_Cl
*M* *_r_* = 890.31
Hexagonal, 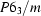

*a* = 10.1969 (1) Å
*c* = 7.28108 (9) Å
*V* = 655.63 (2) Å^3^
*Z* = 2
Synchrotron radiation
λ = 0.998043 Å
*T* = 298 K
Specimen shape: cylinder
40 × 0.7 × 0.7 mm
Specimen prepared at 100 kPa
Specimen prepared at 1258 K
Particle morphology: powder, white

#### Data collection

In-house design diffractometer
Specimen mounting: capillary
Specimen mounted in transmission mode
Scan method: step
Absorption correction: fixed at 0
2θ_min_ = 2, 2θ_max_ = 60°
Increment in 2θ = 0.01°

#### Refinement

*R*_p_ = 0.052
*R*_wp_ = 0.066
*R*_exp_ = 0.047
*R*_B_ = 0.090
*S* = 2.00
Excluded region(s): 2-6° 2θ
Profile function: Pseudo Voigt
225 reflections
16 parameters
Preferred orientation correction: none

### 

Data collection: local software; cell refinement: *CELREF* (Laugier & Bochu, 2003 ▶) and *GSAS* (Larson & Von Dreele (2004 ▶); data reduction: local software; method used to solve structure: coordinates taken from a related compound; program(s) used to refine structure: *GSAS* and *EXPGUI* (Toby, 2001 ▶); molecular graphics: *VESTA* (Momma & Izumi, 2008 ▶); software used to prepare material for publication: *publCIF* (Westrip, 2009 ▶).

## Supplementary Material

Crystal structure: contains datablocks global, I. DOI: 10.1107/S1600536809005054/br2096sup1.cif

Rietveld powder data: contains datablocks I. DOI: 10.1107/S1600536809005054/br2096Isup2.rtv

Additional supplementary materials:  crystallographic information; 3D view; checkCIF report

## Figures and Tables

**Table:** 

Sr1—O1	2.49 (2)
Sr1—O2^i^	2.59 (2)
Sr1—O3^i^	3.01 (1)
Sr2—O2^ii^	2.53 (2)
Sr2—O3^iii^	2.44 (1)
Sr2—O3^iv^	2.94 (1)
Sr2—O1^v^	3.02 (2)
Sr2—Cl1^iv^	3.156 (3)
As1—O3	1.57 (1)
As1—O1	1.72 (2)
As1—O2	1.70 (2)

**Table:** 

O3—As1—O3^vi^	121 (1)
O3—As1—O1	105.8 (7)
O3—As1—O2	106.3 (6)
O1—As1—O2	112 (1)

Symmetry codes: (i) 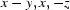; (ii) 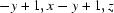; (iii) 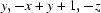; (iv) 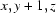; (v) 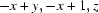; (vi) 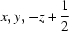.

**Fig. 1.**
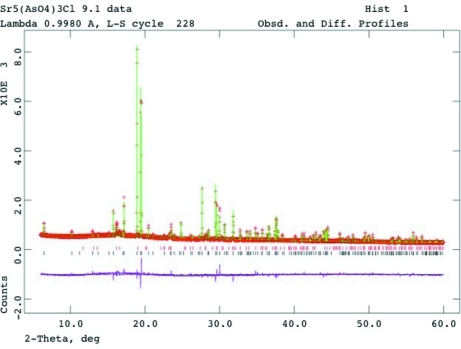
Rietveld difference plot for the multi-phase refinement of Sr5(AsO4)3Cl and SrCO3. The red crosses, and green and pink lines show respectively the observed, calculated and difference plots. Calculated Bragg reflection positions are indicated by black lines for the Sr5(AsO4)3Cl phase and by red lines for the SrCO3 phase.

**Fig. 2.**
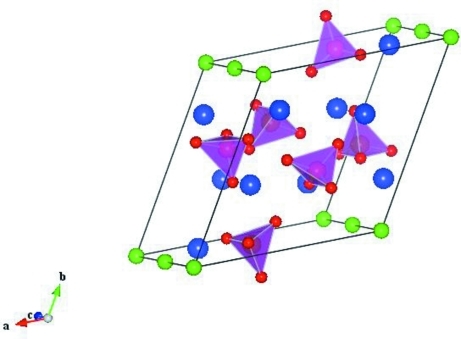
The crystal structure of Sr5(AsO4)3Cl. Pink tetrahedra show AsO4 units with As5+ cations as orange spheres and O2- anions as red spheres. Large blue spheres represent Sr2+ cations and small green spheres Cl- anions.

## supplementary crystallographic information

### Comment

Apatites are minerals and synthetic compounds with general formula *A*_5_(*Y*O_4_)_3_*X*, containing tetrahedrally coordinated *Y*O_4_^3-^ anions (*Y* = pentavalent cation) and a monovalent anion *X* such as F^-^, Cl^-^ or OH^-^. The divalent cations frequently belong to the alkaline earth group, but other cations like Pb^2+^ are also known. For a review of the structures and crystal-chemistry of these materials, see Mercier *et al.* (2005), White & ZhiLi (2003) and Wu *et al.*, (2003). Apatites containing arsenic (As-apatites) are of interest as hosts for storage of arsenic removed from contaminated water (Harrison *et al.*, 2002). Powder diffraction data for the Sr containing As-apatite Sr_5_(AsO_4_)_3_Cl (Kreidler & Hummel, 1970) was indexed in space group *P*6_3_/*m*. Related crystal structures have also been reported for Ca_5_(AsO_4_)_3_Cl (Wardojo and Hwu, 1996) and for Sr_5_(AsO_4_)_3_F and (Sr_1.66_Ba_0.34_)(Ba_2.61_Sr_0.39_)(AsO_4_)_3_Cl (&Dstrok;ordević *et al.*, 2008). The crystal structure of Sr_5_(AsO_4_)_3_Cl in space group *P*6_3_/*m* is reported in the present communication. We recently reported the related crystal structure of Ba_5_(AsO_4_)_3_Cl (Bell *et al.*, 2008).

Table 1 shows refined interatomic distances and angles for the Sr_5_(AsO_4_)_3_Cl structure. The averaged Sr1—O and Sr2—O distances of respectively 2.70 Å and 2.72 Å, compare with Sr1—O and Sr2—O distances in: Sr_5_(AsO_4_)_3_F (&Dstrok;ordević *et al.* 2008) of 2.71 Å and 2.62 Å; 2.71 Å and 2.63 Å for Sr_5_(VO_4_)_3_Cl (Beck *et al.*, 2006); 2.67 Å and 2.62 Å for Sr_5_(PO_4_)_3_Cl (Sudarsanan and Young, 1974); and 2.67 Å and 2.59 Å for Sr_5_(PO_4_)_3_F (Swafford and Holt, 2002). The As—O distances are characteristic for tetrahedral AsO_4_ units. The O—As—O angles deviate significantly from the ideal tetrahedral angle of 109.5°, indicating a strong distortion.

The refined lattice parameters for Sr_5_(AsO_4_)_3_Cl are similar to the previously published parameters of a = 10.18 Å, c = 7.28 Å given by Kreidler & Hummel (1970). Fig. 1 shows the Rietveld difference plot for the present refinement. The crystal structure of Sr_5_(AsO_4_)_3_Cl, showing the isolated tetrahedral AsO_4_^3-^ anions separated by Sr^2+^ cations and Cl^-^ anions, is displayed in Fig. 2.

### Experimental

This work was part of an attempt to synthesize analogues of Pb_5_(AsO_4_)_3_Cl (mimetite) with Pb^2+^ substituted by alkaline earth cations. All starting materials were well crystallized solids. Pb_5_(AsO_4_)_3_Cl was precipitated by titration of 0.1*M* Na_2_HAsO_4_ into a well stirred, saturated PbCl_2_ solution at room temperature (procedure modified from methods of Baker (1966) and Essington (1988)). The molar ratio of Pb:As was slightly greater than 5:3, allowing for excess PbCl_2_ during the precipitation. A very fine-grained pure solid formed immediately, which was then separated, washed, and dried. Typically, five de-ionized water washes were needed to reduce the conductivity of the wash water to < 50 µS^.^cm^-1^. Sr_5_(AsO_4_)_3_Cl was successfully synthesized by ion exchange of Pb_5_(AsO_4_)_3_Cl with molten SrCl_2_ at 1258 K (modified from the method given by Kreidler & Hummel (1970)). Two fusions were required to completely eliminate formation of Pb containing solid solutions and to yield the Pb free title compound. Excess metal in the form of SrCl_2_ was removed from the solids by repeated washing with de-ionized water followed by centrifugation and filtration to separate the solid from the solution.

### Refinement

The main Bragg reflections of the high resolution synchrotron X-ray powder diffraction pattern could be indexed in space group *P*6_3_/*m* with similar lattice parameters to those of the published powder diffraction data (Kreidler & Hummel, 1970). Some broad and weak Bragg reflections were matched by the pattern of SrCO_3_ in space group *Pmcn*.

Initial lattice parameters for the two phases were refined using *CELREF* (Laugier & Bochu, 2003). The *P*6_3_/*m* crystal structure of Ba_5_(AsO_4_)_3_Cl (Bell *et al.*, 2008) was used as a starting model for the Rietveld (Rietveld, 1969) refinement of the structure of Sr_5_(AsO_4_)_3_Cl. The crystal structure of strontianite (de Villiers *et al.*,  1971) was used as a starting model for refinement of the structure of SrCO_3_. Isotropic atomic displacement parameters were used for both phases. For the Sr_5_(AsO_4_)_3_Cl phase soft constraints were used for the As—O distances in the AsO_4_ tetrahedral units. These distances were restrained to those for mimetite (Dai *et al.*, 1991). For the SrCO_3_ phase only the coordinates and the atomic displacement parameters for Sr were refined, the C and O coordinates were fixed to those in the starting model and the C and O atomic displacement parameters were fixed at zero. Proportions of the two phases were refined as 76.6 (1) wt.% Sr_5_(AsO_4_)_3_Cl and 23.4 (1) wt.% SrCO_3_.

### Crystal data

**Table:**

Sr_5_(AsO_4_)_3_Cl	*D*_x_ = 4.510 (1) Mg m^−^^3^
*M**_r_* = 890.31	Synchrotron radiation, λ = 0.998043 Å
Hexagonal, *P*6_3_/*m*	*T* = 298 K
*a* = 10.1969 (1) Å	Particle morphology: powder
*c* = 7.28108 (9) Å	white
*V* = 655.63 (2) Å^3^	cylinder, 40 × 0.7 mm
*Z* = 2	Specimen preparation: Prepared at 1258 K and 100 kPa

### Data collection

**Table:**

In-house design diffractometer	Data collection mode: transmission
Radiation source: Synchrotron	Scan method: step
Si(111) channel-cut crystal	2θ_min_ = 6°, 2θ_max_ = 60°, 2θ_step_ = 0.01°
Specimen mounting: capillary	

### Refinement

**Table:**

*R*_p_ = 0.052	Profile function: Pseudo Voigt
*R*_wp_ = 0.066	16 parameters
*R*_exp_ = 0.047	0 restraints
*R*_Bragg_ = 0.090	4 constraints
*R*(*F*^2^) = 0.33148	(Δ/σ)_max_ = 0.001
χ^2^ = 3.992	Background function: Cosine Fourier Series
5801 data points	Preferred orientation correction: None
Excluded region(s): 2-6° 2θ	

### Special details

**Table:**

Experimental. Absorption correction fixed at zero, all attempts to refine this term in GSAS were unsuccessful so this term was fixed at zero. CELREF was used for initial lattice parameter determinations before Rietveld refinement. Lattice parameters from GSAS refinement are quoted in the paper.

### Fractional atomic coordinates and isotropic or equivalent isotropic displacement parameters (Å^2^)

**Table:**

	*x*	*y*	*z*	*U*_iso_*/*U*_eq_	
Sr1	0.33333	0.66667	0.008 (1)	0.0246 (9)	
Sr2	0.2496 (5)	0.9936 (6)	0.25	0.0246 (9)	
As1	0.4057 (5)	0.3718 (5)	0.25	0.029 (2)	
O1	0.337 (3)	0.496 (2)	0.25	0.015 (4)	
O2	0.598 (2)	0.464 (2)	0.25	0.015 (4)	
O3	0.354 (2)	0.284 (2)	0.063 (2)	0.015 (4)	
Cl1	0.0000	0.0000	0.0000	0.031 (5)	

### Geometric parameters (Å, °)

**Table:**

Sr1—O1^i^	2.49 (2)	Sr2—O3^vi^	2.44 (1)
Sr1—O1^ii^	2.49 (2)	Sr2—O3^vii^	2.94 (1)
Sr1—O1	2.49 (2)	Sr2—O3^viii^	2.94 (1)
Sr1—O2^iii^	2.59 (2)	Sr2—O1^ii^	3.02 (2)
Sr1—O2^iv^	2.59 (2)	Sr2—Cl1^viii^	3.156 (3)
Sr1—O2^v^	2.59 (2)	Sr2—Cl1^ix^	3.156 (3)
Sr1—O3^iv^	3.01 (1)	As1—O3	1.57 (1)
Sr1—O3^iii^	3.01 (1)	As1—O3^x^	1.57 (1)
Sr1—O3^v^	3.01 (1)	As1—O1	1.72 (2)
Sr2—O2^i^	2.53 (2)	As1—O2	1.70 (2)
Sr2—O3^iv^	2.44 (1)		
			
O3—As1—O3^x^	121 (1)	O3—As1—O2	106.3 (6)
O3—As1—O1	105.8 (7)	O3^x^—As1—O2	106.3 (6)
O3^x^—As1—O1	105.8 (7)	O1—As1—O2	112 (1)

Symmetry codes: (i) −*y*+1, *x*−*y*+1, *z*; (ii) −*x*+*y*, −*x*+1, *z*; (iii) *x*−*y*, *x*, −*z*; (iv) *y*, −*x*+*y*+1, −*z*; (v) −*x*+1, −*y*+1, −*z*; (vi) *y*, −*x*+*y*+1, *z*+1/2; (vii) *x*, *y*+1, −*z*+1/2; (viii) *x*, *y*+1, *z*; (ix) −*x*, −*y*+1, *z*+1/2; (x) *x*, *y*, −*z*+1/2.
